# Epidemiology of Injuries in Professional and Amateur Football Men (Part II)

**DOI:** 10.3390/jcm12196293

**Published:** 2023-09-29

**Authors:** Tudor Vladimir Gurau, Gabriela Gurau, Carmina Liana Musat, Doina Carina Voinescu, Lucretia Anghel, Gelu Onose, Constantin Munteanu, Ilie Onu, Daniel Andrei Iordan

**Affiliations:** 1 Faculty of Medicine and Pharmacy, ‘Dunarea de Jos’ University of Galati, 800008 Galati, Romania; gurauvlad123@gmail.com; 2 Department of Morphological and Functional Sciences, Faculty of Medicine and Pharmacy, ‘Dunarea de Jos’ University, 800008 Galati, Romania; gabigurau@yahoo.com; 3 ‘Sf. Apostol Andrei’ Clinical Emergency County Hospital, 800578 Galati, Romania; carinavoinescu@gmail.com (D.C.V.); anghel_lucretia@yahoo.com (L.A.); 4 Clinical Medical Department, Faculty of Medicine and Pharmacy, ‘Dunarea de Jos’ University of Galati, 800008 Galati, Romania; 5 Neuromuscular Rehabilitation Clinic Division, Teaching Emergency Hospital “Bagdasar-Ar-seni”, 041915 Bucharest, Romania; gelu.onose@umfcd.ro; 6 Faculty of Medicine, University of Medicine and Pharmacy “Carol Davila”, 020022 Bucharest, Romania; 7 Department of Biomedical Sciences, Faculty of Medical Bioengineering, University of Medicine and Pharmacy “Grigore T. Popa” Iasi, 700454 Iasi, Romania; constantin.munteanu.biolog@umfiasi.ro; 8 Department of Individual Sports and Kinetotherapy, Faculty of Physical Education and Sport, ‘Dunarea de Jos’ University of Galati, 800008 Galati, Romania; daniel.iordan@ugal.ro; 9 Center of Physical Therapy and Rehabilitation, ‘Dunărea de Jos’ University of Galati, 800008 Galati, Romania

**Keywords:** accidents, football, professional, amateur, men, type, severity, mechanisms, injuries

## Abstract

Background (1): Men’s football is a physically demanding contact sport that involves intermittent bouts of sprinting, jogging, walking, jumping and changes of direction. The physical demands of the game vary by level of play (amateur club, sub-elite and open club or international), but injury rates at all levels of the men’s football game remain the highest of all sports. Objective: The aim of this study is to conduct a systematic review of data from the epidemiological literature regarding the profile, severity and mechanisms of injuries and the frequency of recurrent injuries in professional and amateur football players. Methods (2): A systematic review, according to PRISMA guidelines, was performed up to June 2023 in the databases of PubMed, Web of Science, Google academic, Google scholar and the Diva portal. Twenty-seven studies that reported data on the type, severity, recurrence and mechanisms of injury in professional and amateur men’s football were selected and analyzed. Two reviewers independently audited data and assessed the study quality using the additional and adapted version of the Strengthening the Reporting of Observational Studies in Epidemiology (STROBE) statement and the Newcastle Ottawa Scale (NOS) to assess risk of bias for the quality of external validity. Results (3): In professional male football players, the mean prevalence of muscle/tendon injuries was 39.78%, followed by joint and ligament injuries—21.13%, contusions—17.86%, and fractures—3.27%, and for amateur football players, the prevalence’s were 44.56% (muscle/tendon injuries), 27.62% (joint and ligament injuries), 15.0% (contusions) and 3.05% (fracture), respectively. The frequency of traumatic injuries was higher in amateur football players (76.88%) compared to professional football players (64.16%), the situation being reversed in the case of overuse injuries: 27.62% in professional football players and 21.13% in amateur football players. Most contact injuries were found in professional footballers (50.70%), with non-contact injuries predominating in amateur footballers (54.04%). The analysis of the severity of injuries showed that moderate injuries dominated in the two categories of footballers; the severe injuries in amateur footballers exceeded the severe injuries recorded in professional footballers by 9.60%. Recurrence proportions showed an inverse relationship with the level of play, being higher in amateur footballers (16.66%) compared to professional footballers (15.25%). Conclusions (4): Football-related injuries have a significant impact on professional and amateur football players and their short- and long-term health status. Knowing the frequency of severe diagnoses, such as strains, tears and cramps of the thigh muscles, ankle ligament sprains and hip/groin muscle strain requires the establishment of adequate programs to prevent them, especially in amateur football players, who are more prone to serious injuries.

## 1. Introduction

Football is a sport of wide interest due to the large number of athletes involved, from young people to professionals of all ages (about 270 million). For 110,000 athletes, it is a profession; for about 38 million players, it is a team game organized in leagues and competitions; and for an estimated 226 million others, it is enjoyable exercise for fitness and health [1]. Performance in football depends on a variety of individual, technical and tactical skills and the interaction between different players within the team. Players on successful teams exhibit superior physical capabilities, such as specific physiological and neuromuscular abilities. Football is a fast, high-energy sport that involves multiple motor actions such as sprinting, changing direction, ball-specific skills, jumping and player-to-player contact [2], which associates it with a high risk of injury. Injuries occur during play and practice especially due to the combination of high speed and full contact. Authors of various studies have shown that the incidence of injuries in professional football varied from 0.5 to 45 injuries per 1000 h of exposure to matches and training [3,4,5,6,7]. Most football injuries frequently affect the knee and ankle joints, and the muscles and ligaments of the thigh and calf. In professional male football players, the most commonly affected region is the lower extremity with an incidence from 61 to 90% [7,8,9,10,11,12]. In general, it can be observed that around 66% of football injuries are classified as traumatic, while the remaining third (approximately 27–33%) are attributed to overuse, as supported by various sources [4,8,11]. Two-thirds of traumatic injuries are contact injuries, of which 12–28% are caused by rough play. In particular, non-contact injuries represent 26–58% of all injuries [12]. The most common types of injuries were muscle/tendon injuries, joint and ligament injuries, and contusions. Muscle injuries are a substantial problem for players and their clubs. They account for almost a third of all time-loss injuries in men’s professional football, and 92% of all injuries affect the 4 major muscle groups in the lower limbs—hamstrings (37%), adductors (23%), quadriceps (19%) and calf muscles (13%) [13]. Ankle injuries accounted for 10–18% of all injuries in high-level football [13,14,15,16,17,18,19]. Sprains accounted for 51–81% of all ankle injuries, mainly affecting the lateral ligaments [14,17].

While many studies have focused on the incidence of injuries in professional football [4,20,21], far too few have reported data on injury rates, the type, mechanisms and recurrence of injuries in male amateur football players [22,23,24].

The aim of the current review is to compare between professional and amateur footballers the type and severity of injuries; mechanism of injury; overuse injuries vs. traumatic injuries; contact injuries vs. non-contact injuries; recurrent injuries vs. new lesions; national leagues vs. international tournaments; level of play.

## 2. Method

The method, search strategy and eligibility criteria, data extraction, quality and risk of bias assessment, statistical analyzes and descriptive characteristics of the studies are described in the article “Epidemiology of injuries in professional and amateur football—A systematic review (Part I)”, in the Supplementary Materials (Tables S1–S6, Figure S1) and in the PRISMA flow chart [25]. Our review was conducted in accordance with the Protocol Guidelines for Systematic Reviews of Observational Studies in Epidemiology, PRISMA (Preferred Reporting Items for Systematic Reviews and Meta-Analyses) [25].

### 2.1. Search Strategy and Eligibility Criteria

Analyzed studies were identified in the electronic databases of PubMed, Medline and Google scholar, Diva Portal and Google, and published until June 2023. The key words entered were: epidemiology injuries men’s football; professional and amateur football; location, type and the severity of the injuries. The search and selection of studies was performed by 2 co-authors. Disagreements between the 2 raters were resolved by discussion. Inclusion criteria were: prospective and retrospective longitudinal research studies and original studies, systematic reviews and meta-analyses; studies that included professional or amateur footballers (male) aged over 18 years for professionals or over 17 years for amateur footballers; footballers active in a senior men’s football league (club or elite amateur club) or international senior men’s football tournament (match between two national teams); studies that provided information on the location, type and severity of lesions; studies that expressed lesions as percentages according to location, type and severity or provided sufficient data to allow their calculation as a percentage. Additional criteria: Studies must be full-text articles or abstracts published in peer-reviewed journals before June 2023 and published in English [25].

### 2.2. Quality and Risk of Bias Assessment

The reporting quality of the included studies was assessed using an adapted version of the Strengthening the Reporting of Observational Studies in Epidemiology (STROBE) statement. In this sense, 11 STROBE criteria were used and designed to assess the quality of the studies included in the systematic review. Items in the STROBE statement were scored between 0 and 11 points, with a maximum score of 11 indicating that the article meets the requirements for a high-quality publication [25]. A version of the Newcastle Ottawa Scale (NOS) was used to assess the risk of bias for external quality validation. The proposed criteria included 8 items. This scale uses a star rating system to indicate the quality of a study. The maximum score of 8 stars indicates the lowest risk of bias. Quality and risk of bias assessment were independently assessed by two co-authors, with disagreements resolved by consensus [25].

### 2.3. Statistical Analyses

All statistical analyzes were performed with Windows Excel 2010. Descriptive data such as percentage lesion characteristics were calculated as mean values and standard deviations (±SD). Where injury severity, type and mechanisms were not expressed as percentages, they were calculated based on data available in the studies.

## 3. Results

### 3.1. Descriptive Characteristics of the Studies

According to Gurau et al. [25], 1525 references were identified by using the search strategies, of which 720 were excluded at the first check as duplicates (47.2%). Of the remaining 805 (52.8%), 704 (46.2% total) were eliminated after reading the titles and abstracts. They referred to injuries in other sports (*n* = 214; 14.0%); in children, adolescents and young athletes U-17 (*n* = 257; 15.5%); in women’s football (*n* = 161; 10.6%); as well as other subjects (*n* = 72; 4.7%). The remaining full-text articles and some conclusive abstracts (*n* = 101; 6.6%) were screened for eligibility, referring to adult professional and amateur football players. Of these (*n* = 55; 3.6%), studies did not report injury incidence (general, training and match); they defined injury differently from injuries/1000 h of exposure; they did not express the severity, location and type of injuries as a percentage, nor did they provide sufficient data to allow their calculation. The search process resulted in 46 articles (36 studies of professional football and 10 of amateur football) that met the inclusion criteria. These studies were conducted between 1990 and June 2023 for professional athletes and between 2012 and June 2023 for amateurs. Eligible studies included professional and amateur football players, participants in world tournaments, continental tournaments and professional leagues from different countries (England, Switzerland, Qatar, Iran, Brazil, USA, Spain, Netherlands, Norway, Italy, Germany, Portugal, Ireland and Iceland).

### 3.2. Type of Injuries in Professional Football

Twenty-seven studies reported the prevalence of injuries classified into muscle and tendon injuries, ligament and joint injuries, concussion, fractures, abrasions and lacerations, and peripheral and central nervous system injuries (Table 1). Muscle strain/tear and tendon injuries accounted for 39.78 ± 11.8% of the total injuries recorded. Reported data varied from study to study, with ranges of variation ranging from 19.0% [26] to 67.24% [27]. Muscle injuries were followed by ligament and joint sprains, which on a mean represented 21.13 ± 4.14%, with a range of variation of 13.1% [28]–35.7% [29] and contusions with a mean value of 17.86 ± 3.82% and a range of variation between 5.61% [27] and 30.0% [30]. Three studies [31,32,33] did not report the prevalence of contusions. The prevalence of fractures was much lower compared to muscle, joint and contusion injuries, with the mean value being 3.27 ± 0.89% (1% [30]–8.9% [34]). Árnason et al. [35] and [7,27,31,32,36,37] did not show fractures in their studies. Mean values for lacerations and peripheral and central nervous system injuries were 2.42 ± 1.42% and 1.3 ± 1.16%, respectively, and calculated for 12/27 and 7/27 studies, respectively.

### 3.3. Injury Type in Amateur Football Players

**Figure 1:**
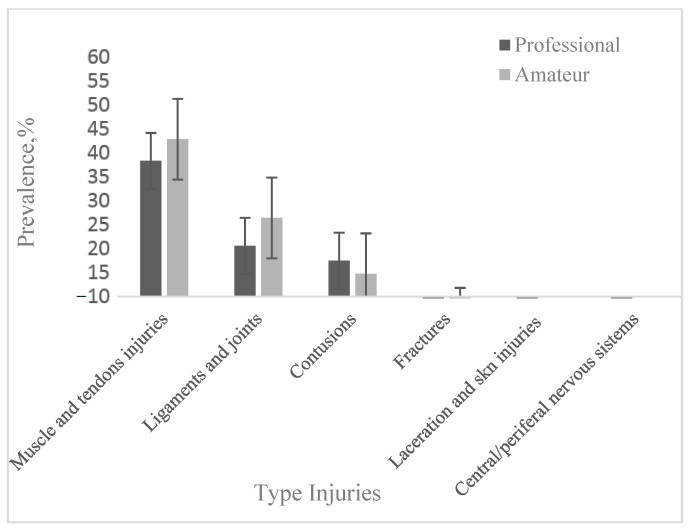
Prevalence of lesions depending on their type.

In amateur football players, muscle/tendon injuries also prevailed (Table 2); the mean value of the studies was 44.56 ± 12.57%, with a range of variation between 17.4% [44] and 65% [18,26]. For joint and ligament injuries, a mean of 27.62 ± 7.18% was recorded (10.0% [19]–33.0% [26]). The prevalence of contusions was relatively lower, with a mean value of 15.04 ± 4.15% (6.8% [18]–23.5% [44]). Fractures ranged from 1.0% [35,44] to 6.4% [44], with the mean value being 3.05 ± 2.58%. Only Herrero et al. [44] reported skin lesions/lacerations (2.4%) and central/peripheral nervous system injuries (0.1%). A pooled analysis of studies by injury type shows differences between professional and amateur football players. Muscle/tendon and ligament/joint injuries were more frequent in amateur footballers compared to professional footballers, with 4.78% and 6.49%, respectively. Contusions were 2.86% more numerous in professional footballers than in amateur ones. The observed fracture data show a difference of only 0.22% in professional versus amateur footballers (Figure 1). The very low number of reports of skin lesions/lacerations and central/peripheral nervous system injuries in amateur footballers made it impossible to compare the data with those from professional footballers.

### 3.4. The Mechanism of Accidents in Professional Football Players

Regarding the mechanism of injury production, 27 studies (Table 3) reported data either on traumatic and overuse injuries (24 studies), or with contact or non-contact (12 studies). Seventeen studies reported the frequency of recurrent injuries. Mean values were calculated taking into account only studies that reported data on the considered parameter. The most frequent injuries were traumatic, representing the mean 64.16 ± 15.34% (21.5% [27]–94% [34]), with the rest being caused by overuse—35.60 ± 15.22% (6–78.6%). Of all injuries, 50.70 ± 11.56% were contact, and 49.30 ± 11.56% were non-contact. A pooled analysis of studies resulted in a mean prevalence of recurrent injuries of 15.26 ± 5.81%.

### 3.5. The Mechanism of Accidents in Amateur Football Players

In relation to professional footballers, traumatic injuries were more frequent, with a mean prevalence of (76.88 ± 9.05%) compared to (64.16 ± 15.34%), noting a difference of 12.72% (Table 4). Correspondingly, overuse injuries were 12.74% fewer in amateur than professional footballers (22.86 ± 8.74 vs. 35.60 ± 15.22%). Non-contact injuries in professional footballers exceeded non-contact injuries in amateur footballers by 5.74% (55.04 ± 16.1% vs. 49.30% ± 11.56%), with the situation being reversed in the case of contact injuries (45.96 ± 16.1 vs. 50.70 ± 11.56%). The mean value of recurrent injuries in amateur footballers was relatively similar to that found in professional footballers: (16.66 ± 10.25%) vs. (15.26 ± 5.81%).

For the studies that presented complete values on injury mechanisms, the correlation coefficient (correl) was calculated which showed a weak positive association between traumatic and contact injuries in professional football players (0.327) and moderate association in amateur football players (0.748). Overuse injuries showed negative associations with contact injuries in both categories of football players (−0.327 and −0.748, respectively).

### 3.6. Severity of Injuries: Professional Football Players

The injuries were divided into four severity categories according to the number of days absent: minimal (1–3 days), mild (4–7 days), moderate (8–28 days) and severe (>28 days) (Additional file S2; Part [25]). Data on injury severity in professional football players are presented in Table 5. Twenty-seven studies reported injury severity as a percentage or reported the data needed to calculate it. Calculated values have been marked with an asterix [7,14,18,19,26,27,30,40,43,47]. The most frequent lesions were moderate, with a mean value of 33.57 ± 8.37% and a wide range of variation, between 13.64% [19] and 69.3% [52], followed by minimal damage with a mean value of 27.94 ± 11.93% (range of variation 7.8% [52] and 60.5% [43]) and mild lesions with a mean of 24.52 ± 6.71% (range of variation between 9.09% [19] and 60.0% [16]). Serious injuries were less frequent, with the mean value being 13.32 ± 5.59% and the range of variation being between 3.6% [52] and 41.6% [44].

### 3.7. Severity of Injuries: Amateur Football Players

**Figure 2:**
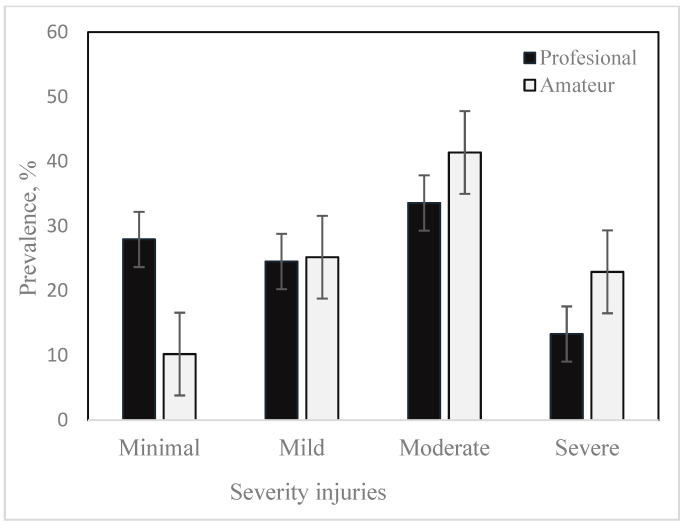
Severity injuries in football.

Regarding injury severity in amateur football players, six studies (Table 6) were included in the pooled analysis [14,22,23,32,50,53]. As in the case of professional football players, the most frequent injuries were moderate, with a mean value of 41.38 ± 7.56% and a range of variation between 23.3% [22] and 64.7% [32]. In second place were mild injuries, with a mean value of 25.19 ± 7.56% and a range of variation between 13.7% [32] and 40.8% [22], followed by minimal lesions with a mean of 10.21 ± 7.77%, with percentages reported in studies varying between 0% [32] and 32% [22]. Severe lesions had a prevalence of 22.92 ± 9.0% and a range of variation between 3.9% [22] and 46% [32]. Van Beijsterveldt et al. [53] reported for the Intervention Group 0.5% career-ending injuries and for the Control Group 0.5% minor injuries and 1.4% total loss of ability to play football. Severe and moderate injuries were more common in amateur footballers than in professionals, with the mean values being 1.72 and 1.23 times higher, respectively, while minimal injuries prevailed in professional footballers—27.94 ± 11.93% versus 10.21 ± 7.77% for amateurs. The share of mild injuries was similar in the two categories of footballers: 25.19% versus 24.52% (Figure 2). Incomplete data prevented the calculation of joint estimates for injury burden among professional and amateur football players.

## 4. Discussions

### 4.1. The Type of Injury

In our study, the most common types of injuries in professional football players were muscle and tendon injuries (prevalence 39.78 ± 11.8%), joint and ligament injuries (21.13 ± 4.14%) and contusions (17.86% ± 3.82). In amateur football players, the prevalence of muscle injuries was 44.56 ± 12.57% and that of joint and ligament injuries was27.62% ± 7.18; the prevalence of contusions was lower (15.0 ± 4.15%). Fractures were less frequent with a prevalence of 3.27 ± 0.89% in professional footballers and 3.05 ± 2.58% in amateurs. Lacerations and abrasions (2.42 ± 1.42%) and central/peripheral nervous system injuries (1.3 ± 1.16%) were reported only in professional football players (Table 1 and Table 2).

Our data on muscle injuries are consistent with the results of other studies stating that muscle injuries are the most common in football [54,55,56,57], representing more than 30% of injury types. Árnason et al. [58] stated that muscle strains (29%), ligament sprains (22%) and contusions (20%) were the most common types of injury. The frequency of re-injury was significantly high, with 44% of strains and 58% of sprains recorded as re-injuries.

Palazon et al. [20] found, for young male football players, that muscle/tendon injuries were the most frequent (IIR = 1.90/1000 h), followed by joint and ligament injuries (IIR = 0.97/1000 h) and contusions (IIR = 0.84/1000 h); bone fracture and stress, central/peripheral nervous system injuries, lacerations and skin injuries were the less common injury types. In young female football players, joint and ligament injuries (IIR = 2.36 injuries/1000 h) were the most common, followed by muscle/tendon injuries (IIR = 2.01 injuries/1000 h) and contusions (IIR = 0.93 injuries/1000 h). Bone stress fractures and injuries (IIR = 0.27 injuries/1000 h) were less common, and skin lacerations or central/peripheral nervous system injuries were not recorded. At the muscle and tendon level (two fibrous tissues that connect muscles to bones), the injuries consisted of strains/tears, which occur when the tissue is overstressed, mainly due to sudden acceleration or deceleration. Over 90% of muscle injuries affected the four major muscle groups of the lower extremity: hamstrings, adductors, quadriceps and gastrocnemius [31]. Several studies have found that hamstring injuries predominate [18,19,33,47]. Roe et al. [6] indicated for these injuries a prevalence of 21% and specified that the biceps femoris (84%) was more frequently injured than the semimembranosus (11%) and semitendinosus (5%) muscles. In elite players, Ekstrand et al. [59] reported prevalences ranging from 12 to 16% of time-wasting injuries. Factors that increase the risk of hamstring muscle injury are: sports that involve extreme strain, running or sprinting; previous hamstring muscle injury; reduced flexibility; or muscle imbalance. Hamstring overuse injuries are common, especially in sports such as football, basketball and tennis, where running is combined with quick starts and stops. Hamstring injuries compromise individual performance and team success in many sports [31,59,60,61,62,63].

Quadriceps strains occur especially in sports that require repetitive kicking and sprinting efforts and are common in football in its various forms around the world [64,65,66,67]. Quadriceps muscle strains are prevalent in the Australian Football League (AFL), with each team reporting 1–2 new quadriceps strain injuries per season [66]. There is a greater risk of hamstring injuries during the first season, while rectus femoris strains (29%) were more common than biceps femoris muscle injuries (11%) in the Premier League pre-season English and Australian Football League [65]. In contrast, Ekstrand et al. [13] found that quadriceps muscle strains were fairly constant throughout the season. Football players with quadriceps injuries miss more games than those with hamstring and groin injuries, and re-injury rates are high (17%).

Ankle sprains are the most common pathology in ankle injuries, accounting for 51–81% of all football-related ankle injuries [14,15,16,17,68,69]. Ankle sprains affect the lateral ligaments, the majority occurring during contact with players (59%), with the exception of goalkeepers, where 79% occur in non-contact situations [70,71]. Jain et al. [72] indicated a 28.6% recurrence of anterior talofibular ligament injury.

The incidence of knee injuries during competition is 15–19% of all injuries. Of these, 35–37% are strains, 20–21% are sprains and 16–24% are contusions. Knee injuries represent 58% of all major injuries [59,73]. The most common knee injuries sustained in football include the anterior cruciate ligament (ACL; 14.2%), medial collateral ligament (MCL; 23%), tears [74] and meniscal tears. The anterior cruciate ligament (ACL) of the knee is at risk of injury during sports involving cutting, jumping and pivoting movements, such as in American football and other sports [75]. They appear in sports involving pivoting, such as football, basketball and handball in European teams, as well as gymnastics and alpine skiing. These can range from mild (small tears/sprain) to severe (when the ligament is completely torn). Both contact and non-contact injuries can occur [76], with non-contact tears being more common when the limb is not in contact and combined with valgus and internal rotation trauma. There is evidence suggesting that the occurrence of ACL injuries is more prevalent among women compared to males, with incidence rates ranging from 2.4 to 9.7 times greater in female athletes engaged in equivalent sports [77,78,79].

Acute ACL rupture is a common trauma, with an incidence of up to 84/100,000 people in the USA and 78/100,000 people in Sweden [80]. According to the findings of Rothenberg et al. [74], the yearly prevalence of the anterior cruciate ligament (ACL) damage among female football players ranges from 0.5% to 6.0%, whereas, among male football players, it ranges from 0.6% to 8.5%.

In professional sports, epidemiological studies of concussion have been reported in Australian Rules Football [81,82] and globally in football [83,84], Major League Baseball [85], National Basketball Association [86], National Hockey League [87] and in rubies [88]. Some studies that used publicly available data reported rates of 0.66 concussions/per game or 1.6 concussions/per game [81,89,90,91,92,93]. Variation in time frame and methodology led to the variation in reported incidences. Most head injuries in men’s professional football are caused by head-to-head and elbow-to-head contact [94]. Within our study, only a few articles addressed the subtypes of football injuries [18,19,47,48,95,96].

### 4.2. Mechanism of Injury

In our systematic review, 25 studies (Table 3) of professional football players and 5 studies of amateur football players (Table 4) provided data comparing traumatic (acute) injuries with overuse injuries. The majority of football-related injuries had a traumatic mechanism, with a prevalence of 64.16 ± 15.34% in professionals and 76.88 ± 9.05% in amateurs that was 1.8 times higher and 3.36 times higher, respectively, compared to overuse injuries (35.60 ± 15.22%—professionals and 22.86 ± 8.74%—amateurs). In relation to the mechanism of injury, 15 professional and 5 amateur studies reported contact and non-contact injury data among professional and amateur male football players. In professional football players, a higher prevalence of contact injuries was recorded (50.70 ± 11.56 vs. 49.30 ± 11.56%), while in amateur football players, the prevalence was higher for non-contact injuries (55.04 ± 16.1 vs. 45.96 ± 16.1%). Several authors reported that more than two-thirds of football injuries are traumatic (67–80%), with the rest (33–20%) being caused by overuse [4,11,97]. Moreover, about two-thirds of traumatic injuries are contact injuries, of which 12–28% are caused by foul play. Non-contact injuries accounted for 26–58% of all injuries [14,15,97,98], which are results that are close to the data presented by us. Sprouse et al. [95] found a higher prevalence of contact injuries in senior football players than non-contact injuries (54% vs. 40%), indicating a higher match vs. training prevalence of contact injuries (63 vs. 40% in seniors and 70 vs. 40% in young people), as in the case of traumatic injuries. The situation is similar for women.

In elite players, contact injuries accounted for 33–42% of all acute injuries [14,15]. Only Luthje et al. [73] found a higher proportion of contact injuries (79%). Studies of lower level players reported that 55–59% of acute injuries were contact injuries, while the comparable percentage for junior players was 42–53% [14,99,100].

In young male football players, Palazon et al. [20] state that the incidence rate for traumatic injuries (5.50 injuries/1000 h) was higher than for overuse injuries (1.10 injuries/1000 h), the incidence ratio being higher than what we found in male amateur footballers. Similar to men, in young female football players, the incidence rate for traumatic injuries (4.55 injuries/1000 h) was higher compared to that for overuse injuries (1.56 injuries/1000 h). Moreover, for young footballers of both sexes, the rates of incidence for non-contact injuries were superior to those with contact (3.48 and 2.39 injuries/1000 h vs. 2.77 and 1.92 injuries/1000 h).

In recent years, increased research has focused on overuse injuries among athletes involved in various competitive sports, finding a prevalence of 43–46.2% in volleyball [101,102,103]; 26.2–29.3% rowing youths [104,105]; 82.6% in professional and amateur golf [106]; 50% in basketball, 21.7% in field hockey; 25% in football [107]. For basketball, Dick et al. [108] reported the prevalence of contact injuries being 52.5% for matches and 43.6% for training, with values higher than non-contact injuries being 22.3% and 36.3%, respectively.

### 4.3. Severity of Injuries

Eligible studies that reported injury severity data in professional and amateur football players are listed in Table 5 and Table 6. The aggregate prevalences of minimal injuries were 27.94 ± 11,93% in professionals and 10.21 ± 7.77% in amateurs; mild—24.56 ± 6.71% vs. 25.19 ± 7.56%; moderate—33.57% ± 8.37 vs. 41.38 ± 7.56%; severe—13.32 ± 5.59% vs. 22.92 ± 9.0%. Sprouse et al. [97] reported a prevalence of minor injuries in international football: 60% for senior players and 59% for youth football players, with 27% vs. 28% for moderate injuries and 12% vs. 9% for severe injuries, which also indicated 2% vs. 4% of major injuries. Those authors found no significant difference in the distribution of injuries between the match and practice, between seniors and youth for overall injury severity, injury severity during the match or injury severity at practice. The prevalences reported by [21] for female football players with 18% minimal, 20% mild, 40% moderate and 21% severe injuries are closer to the values we reported for male amateur football players vs. professional ones. The same finding is in the case of the results published by [109], who reported that in the first division of women’s football in Spain, 16% of injuries were minimal, 22% were mild, 40% were moderate and 23% were severe. In youth football players, most injuries were of minimal severity (1–3 days lost), but moderate injuries (incidence of 1.7 injuries/1000 h for men and 1.5 injuries/1000 h for women) and severe injuries (0.8 injuries/1000 h for men and 1.3 injuries/1000 h for women), indicated in the meta-analysis by [20], may be a cause for concern.

The most serious injuries in football are bone injuries (tibia, fibula and metatarsal fractures), major ankle breaks or sprains, muscle injuries and those affecting ligaments or tendons. The rupture of the anterior cruciate ligament, tear of the medial lateral ligament and tear of the medial meniscus mark a football player’s career for life. Mild injuries with an absence of less than 7 days were mainly represented by traumatic injuries of the lower extremity, such as contusions or joint capsular and ligamentous injuries or painful overuse syndromes. The moderate ones were mainly structural muscle injuries in the thigh and pain syndromes related to the groin area, while the injuries with the longest absence were those affecting the knee [110]. The results of our study showed that most injuries in male professional and amateur football players with a traumatic or non-contact mechanism are preventable. The implementation of neuromuscular training (NMT) programs can have positive effects on the incidence of injuries in adults [111,112,113,114]. A recent meta-analysis found that a football-specific NMT program reduced injury rates by 20–50% [114]. Regarding ankle injuries, neuromuscular and proprioceptive intervention programs have been found to decrease injury risk by 35–50% in adult sports populations [111,112,113]. Similar effects have been reported for young athletes. Two meta-analyses demonstrated a risk reduction for lower extremity injuries of approximately 25–35% [113,114].

### 4.4. New Versus Recurrent Lesions

The studies included in our review that reported the frequencies of recurrent injuries [115,116] are shown in Table 3 for professional football players (21 studies) and in Table 4 for amateur football players (6 studies). The cumulative frequency of re-injury for professional footballers was 15.26 ± 5.81%, 1.40% lower than that recorded for amateur footballers (16.66 ± 10.25%). The results we obtained are consistent with those reported by [19], who indicated a recurrence of 16.6% for top-level football players; however, this was significantly different from the recurrence in elite (25%) and amateur football players (35.1%). The recurrence occurred in less than two months in all three categories of footballers. This may reflect a premature return to training/play and incomplete or inadequate rehabilitation. Those authors reported higher recurrent injury incidence rates in the match compared to training (3.22/3.72/4.36 vs. 0.58/1.52/1.54/1000 exposure hours for top level/elite/amateurs). The incidence of recurrent lesions was lower compared to the incidence of new lesions (1.3 vs. 7.0 lesions/1000 h of exposure) [19]. For adult female football players, Lopez et al. [117] indicated a lower incidence of recurrent lesions (1.8/1000 h) than the incidence of new lesions (4.6/1000 h of exposure). Rates for recurrent injuries in youth football players were lower than those for new injuries (0.8 injuries/1000 h for men and 1.4 injuries/1000 h for women) vs. 5.9 injuries/1000 h for men and 5.1 injuries/1000 h for women, respectively) [20]. Van der Horst [118], in an analysis of potential risk factors such as age, BMI body weight (BMI), injury history, “The FIFA11” intervention, playing position and surface, injured leg (dominant vs. non-dominant) and total exposure, found that these factors were not significantly associated with an increased risk of recurrent injury. The frequency of recurrent lesions described in this study of 12.9% is in accordance with previously published data on specific types of recurrent lesions. Recurrence incidence between 13.9 and 63.3% was reported for hamstring injuries, while the incidence of recurrent ankle injuries varied between 3 and 34% [118,119,120]. Three specific sites of recurrent injury were identified, with the hamstring (26%), ankle (23%) and knee (14%) being the most common sites of recurrent injury. The recurrence of muscle injuries depended on the type of muscle and the category of footballers. Hägglund et al. [48] indicated, for hamstrings, a prevalence of 22.7%, 15.7% and 12.5% for top-level, elite and amateur footballers, respectively. In the adductors, the most injuries were in elite footballers (14.2%), followed by top-level players (11.6%) and amateurs (15%). The quadriceps and calf muscles were affected to a lesser extent in top-level players and elites, with amateurs registering a prevalence of recurrent calf injuries of 12.5% [48]. Wiggins et al. [121] estimate that 1 in 4 young athletes (<20–25 years) who sustain an ACL injury and return to high-risk sports will go on to sustain another ACL injury at some point in their career. Recurrence proportions were higher in the second half of the competitive season for all cohorts [48].

### 4.5. Game Level

In our review, several studies have shown differences between elites, sub-elites and amateur adult male players in terms of location, type and severity of injury, incidence of recurrent injury and days lost due to injury. Moreover, the variation of these indicators was noted depending on the country of origin of the football team (climate); type of competition—friendly matches or competitive matches (national, in world cups, European cups and Olympics); season; lawn type. In European male professional football, Waldén et al. [122] reported that teams located in northern Europe, with countries that typically have milder summers and longer winters, had a prevalence of traumatic and overuse injuries that was different compared to teams from southern Europe with Mediterranean climates. Moreover, the incidence of anterior cruciate ligament (ACL) injuries, and, in particular, non-contact ACL injuries, was found to follow a reverse trend, with Mediterranean climate teams experiencing more ACL injuries. The injury severity profile indicates: minimal injuries—30.1 (36.20%); mild—27.1 (28.1%); moderate—32.8 (24.7%); severe—10 (11%). Twenty-two percent (18%) of lesions were early recurrences. Players injured in the first year had a higher risk of injury the following season compared to uninjured players (hazard ratio 2.7), those with a previous hamstring injury, groin injury or knee joint injury were two to three times more likely to suffer an identical injury the following season, while no such relationship was found for ankle sprains. Age was not associated with an increased risk of injury (Hägglund et al. [48]. In sub-elite footballers in Australia, Whalan et al. [123] reported a higher prevalence for muscle and ligament injuries (41% and 26%) and carried the highest injury burden (83 and 80 lost days/1000 h, respectively). The most common injuries were observed in the thigh (22%) and ankle (17%), with the prevalence of hamstring injuries (13%) being the highest. The risk of hamstring and calf muscle injury increases with age and more frequently towards the end of each half, suggesting that fatigue is a risk factor [95]. The severity profile of the injury was: minimum—35%; mild—29%; moderate—28%; severe—8%. Recurrent injuries accounted for 20% of all injuries.

Between South American teams and European teams, Bengtsson et al. (2021) [124] found significant differences in the incidence of ligament injuries in training (0.6 vs. 0.3/1000 h); differences in training culture between South America and Europe could influence injury epidemiology. Asian professional football is characterized by a high rate of ACL tears and hamstring injuries (54.4%) [125], with recurrent lesions having a prevalence of 9.9% [126].

Professional players had a lower incidence of moderate and severe injuries than amateur players, but a higher incidence of minimal injuries. These differences can be explained by the smaller number of players in amateur teams, with fewer options to replace injured or injury-prone players. Thus, amateur players had a higher match exposure per player than professionals by 17% [127]. In the case of minor injuries in the amateur cohort, there is the possibility of their underreporting, due to the reduced contact between medical staff and players (2–3 times a week, during training and matches), which contrasts with the daily contact between medical staff and players in professional football. As a result, some minor amateur injuries may not be recorded due to players recovering from minor injuries in the meantime. Moreover, medical support is less consistent given the economic constraints of amateur teams, leading to delayed diagnoses and suboptimal rehabilitation, incomplete wound healing and/or neglect of minor injuries [19,128].

Each sport has a unique injury profile and risk of injury. Modern football involves continuous and intensive cycles of training and games, which predisposes players to higher injury risks and the most common overuse injuries. Football has been the focus of a number of randomized injury prevention trials with the aim of maintaining health, reducing costs and improving player performance [129,130,131,132,133,134]. The studies focused, in particular, on the prevention of knee sprains [135,136], anterior cruciate ligament injuries [137], hamstring muscle strains in elite football players [138,139] and thigh pain [140].

The programs included strength, balance and mobility training, proprioceptive training for semi-professional and amateur football players, physical exercise, educational intervention programs and supervision of players and coaches.

Cardoso-Marinho et al. [141] consider football players’ perceptions of injury risk and prevention, as well as their recognition of injury risk factors (low muscle strength, lack of fitness, fatigue, overtraining and type/condition of surfaces; injury prevention factors such as warm-up, workload monitoring and strength and conditioning training) to be important.

Strategies to prevent moderate and severe injuries are important issues for professional and amateur football players and require further study in the future.

Comparing injury epidemiology in professional and amateur soccer provides valuable information on the relationship between injury characteristics and player abilities. The recorded differences regarding the type of injuries (Table 1 and Table 2); injury mechanisms (Table 3 and Table 4); and injury severity (Table 5 and Table 6) between professional and amateur soccer players can be explained by a different level of play, team size that can influence injury, injury risk and characteristics, limited number of training sessions per week for amateur soccer players, availability of medical support, differences between studies in terms of research populations and methodology used [7,42]. Training quality standards, development of muscle strength, endurance/coordination and different technical and tactical skills can be reasons for the differences found between the two categories of soccer players. Professional soccer players usually have better physical skills compared to amateur players, probably because of the higher physical demands in a professional soccer match [142]. Cometti et al. [143] note differences regarding knee flexor muscle strength and sprint speed over short distances. Moreover, inadequate recovery is an important causal factor in re-injury [144]. Hagglund et al. [48] found an inverse relationship between the level of play and recurrent injury. Players at top-level clubs showed a lower recurrence rate than those playing at lower level clubs. It is speculated that players at top-level clubs benefit from high-quality rehabilitation and support in return for play and competition, under the ongoing control of medical and physiotherapy teams providing sufficient rehabilitation time, which would contribute to lower rates of recurrence in professional players [48]. Not all amateur soccer players have standard injury prevention and medical and physical therapy support. Differences in injury assessment and variations in the qualitative assessment of injury severity may influence the final classification of injuries.

Regarding the quality of study reporting, the mean STROBE quality scale score was 8.55 ± 0.5 (minimum = 5; maximum = 11), and for the NOS scale, the mean score was 7.25 ± 0.69 (minimum = 5; maximum = 8). No studies were excluded based on the STROBE quality scale and risk of bias [25].

### 4.6. Limitation

The strength of this study is that it provides a general estimate of the type, severity, recurrence and mechanisms of injuries in amateur football compared to professional football. It highlights the poor attention paid to the millions of amateur footballers in terms of analyzing the prevalence and severity of injuries, as well as measures to prevent injuries in this category of footballers.

Several limitations were present in the current review. More eligible studies may not have been identified; this was motivated by the fact that the selection of studies was limited to articles published in English. Although the methodological quality assessments were performed by two independent reviewers, the assessment of study quality remains, however, subjective. The different definitions of the type and severity of injuries and their different expression [prevalence (%) or incidence (injuries/1000 h)] explain the inclusion in the systematic review of a small sample of eligible studies (especially in amateur football players) and reflect the inconsistent methodological approach between studies. We only documented data related to the main types of injuries, and did not address injuries stratified by subcategories (muscle type, joints and ligaments, and concussions). Although time loss is widely used to describe injury severity in epidemiology, the lack of data on the number of days lost to injury in most of the included studies made it impossible to report injury burden. We also did not highlight the prevalence of overuse complaints that do not cause time loss but may affect the athlete’s ability to perform on the football field. The analysis of the type and severity of injuries was not differentiated by the game, training and match phases or by the types of matches (league, national and international competitions). Future research should continue to record sports injury epidemiologic data using standardized methods and measurements to understand the injury profile and establish appropriate injury prevention programs.

## 5. Conclusions

Muscle/tendon injuries, joint and ligament injuries, and contusions were the main types of injuries in both categories of footballers. The prevalence of muscle/tendon injuries in amateur football players exceeded their prevalence in professional football players by 4.78%. On the other hand, joint/ligament injuries and contusions prevailed in professional footballers compared to amateurs, exceeding them by 6.49% and 2.86%, respectively. Similar mean prevalences were recorded for fractures. Moderate injuries were more frequent in amateur footballers, as were severe injuries, the latter being 9.60% more compared to professional footballers; the situation reverses in the case of mild injuries (27.94 vs. 10.21%). In both professional and amateur footballers, the traumatic mechanism prevailed, with a higher mean frequency in amateur footballers (76.88% vs. 64.16%). Amateur football players were affected to a lesser extent by contact injuries compared to the much more competitive professional football players. Recurrence was 1.40% higher in amateur football players.

## Figures and Tables

**Table 1:** Type of injuries in professional football.

Study	Muscle and Tendon Injuries, %	Ligaments and Joints, %	Contusions, %	Fractures, %	Lacerations and Skin Injuries, %	Central/Peripheral Nervous System, %	Other Injuries, %
Árnason et al., 2005 [35]	30.7	18.4	20.5	-	-	-	30.4
Aus der Fünten et al., 2014 [38]:							
Season 2008–2009	41.7 *	21.9 *	18.5 *	5.3 *	2.0 *	-	10.6 *
Season 2009–2010	33.6 *	27.5 *	17.4 *	4.7 *	2.7 *	-	14.1 *
Aus der Fünten et al. 2023 [39]	49.4	16.2	12.9	4.5	1.2	-	15.8
Bayne et al., 2018 [40]	30.4 *	24.2 *	12.1 *	3.0 *	-	6.0 *	24.3 *
Brito et al., 2012 [41]; U-19	41.3 *	25.1 *	22.6 *	2.5 *	-	-	8.5 *
Dupont et al., 2010 [27]:							
Group G1	67.24	17.24	10.35	-	3.45	-	1.72
Group G2	57.95	22.43	5.61	-	2.8	-	11.21
Eirale et al., 2010 [42]	43.6	12.8	15.4	5.1	-	-	23.1
Eirale et al., 2013 [31]	68.6	12.5	-	-	-	-	18.9
Ekstrand et al., 2011a [7]	42.0	18.0	17.0	4.0	0.8 *	0.4 *	17.8 *
Hägglund et al., 2005a [33]							
Denmark	20.5 *	20.3 *	14.4 *	2.5 *	-	-	42.3 *
Sweden	20.0	17.0	17.0	3.0	-	-	43.0
Hägglund et al., 2006 [26]:							
Season 2001	23.5 *	15.0 *	15.5 *	2.8 *	-	-	43.2 *
Season 2002	19.0	17.0	15.3 *	2.7 *	-	-	46.0
Hägglund et al., 2007 [28]: Season 2001	44.25 *	13.85 *	16.4 *	3.2 *	0.90 *	0.1 *	21.30 *
Season 2002	43.2	13.1	15.1	3.2	1.70	0.3	23.40
Season 2003	44.3	17.5	18.3	2.6	1.6	0.2	15.50
Hägglund et al., 2009 [19]:							
Men Under-21, 2006	56	20	18	4	0	-	2
Men Under-21, 2007	28	26	31	3	2	-	10
EURO men 2008	34	25	26	4	2	-	9
Hammes et al., 2015 [32]	64.70	17.65	-	-	-	-	17.65
Hawkins et al., 1999 [14]	42.4 *	20.1 *	18.0 *	3.8 *	1.7 *	-	14.0 *
Jones et al., 2018 [37]	41.2	17.1	13.7	-	-	-	28.0
Lee et al., 2014 [43]	29.0	28.0	30.0	2.0	2.0	-	9.0
Mallo et al., 2011 [30]; Under Elite	20.0	17.0	21.0	1.0	-	-	41.0
Murphy et al., 2012 [44]	51.8	13.2	-	4.4	-	-	30.6
Noya Salces et al., 2014b [45]	56.1	20.4	16.4	4.0	1.1	0.9	1.1
Noya Salces et al., 2014a [46]	53.8	24.4	14.2	2.6	1.7	0.7	2.6
Reis et al., 2015 [29]	5	35.7	-	2.9	-	1.4	1.4
	8.6						
Shalaj et al., 2016 [9]	28.7	21.0	16.5	2.6	2.6	-	28.6
Stubbe et al., 2016 [5]	36.4	18.5	17.8	3.1	-	1.7	22.5
Waldén et al., 2005a [47]	26.0	23.0	16.0	2.0	-	-	33.0
Waldén et al., 2005b [18]	22.1 *	15.94 *	17.1 *	2.8 *	-	-	42.06
Waldén et al., 2007 [34]: EURO 2004	26.7 *	22.2 *	22.2 *	8.9 *	13.3 *	-	6.7 *
U-19 2005	35.3 *	29.4 *	29.4 *	-	-	-	5.90 *
Mean Values ± SD	39.78 ± 11.8	21.13 ± 4.14	17.86 ± 3.82	3.27 ± 0.89	2.42 ± 1.42	1.3 ± 1.16	19.90 ± 11.36

* Calculated value; SD—standard deviation. a. Swedish Super League (SWE); b. UEFA Champions League (UCL); ART—Artificial Turf Field; 12%—slight injuries, Hägglund et al. [19].

**Table 2:** Injury type in amateur football players.

Study	Muscle and Tendon Injuries, %	Ligaments and Joints, %	Contusions, %	Fractures, %	Lacerations and Skin Injuries, %	Central/Peripheral Nervous System Injuries, %	Other Injuries, %
Brito et al., 2012 [41]	41.0	34.0	16.0	1.0	-	-	8.0
Hägglund et al., 2016 [48]	55.0 *	10.0 *	-	-	-	-	35.0 *
Hammes et al., 2015 [32]:							
Group Intervention	49.3 *	25.35 *	-	-	-	-	25.35 *
Group Control	51.0	27.0	-	-	-	-	22.0
Herrero et al., 2014 [49]	17.4	39.9	23.5	8.2	2.4	0.1	8.5
Kekelekis et al., 2023 [22]	65.0	19.4	6.8	1.0	-	-	7.8
Nogueira et al., 2017 [50] U-17 + U-19	52.8	32.3	12.9	2.0	0	0	0
Sousa et al., 2012 [23]	25.0	33.0	16.0	-	-	-	26.0
Mean Values ± SD	44.56 ± 12.57	27.62 ± 7.18	15.0 ± 4.15	3.05 ± 2.58	-	-	16.58 ± 10.51

* Calculated Values; SD—standard deviation.

**Table 3:** Mechanism of accidents in professional football players.

Study	Traumatic Injuries, %	Overuse Injuries, %	Contact Injuries, %	Non-Contact Injuries, %	Recurrent Injuries, %
Arnason et al., 2005 [35]	84.0	16.0	-	-	-
Aus der Fünten et al., 2014 [38]:					
Season 2008–2009	64.9 *	35.1 *	-	-	-
Season 2009–2010	66.4 *	33.6 *	-	-	-
Aus der Fünten et al., 2023 [39]	-	-	31.8	68.2	-
Brito et al., 2012, Total [41]	57.0	43.0	71.0	29.0	-
Dupont et al., 2010 [27]:					
Group G1	27.6	72.4	-	-	8.6
Group G2	21.5	78.5	-	-	15.9
Eirale et al., 2010 [42]	87.2	12.8	41.0	59.0	24.4
Eirale et al., 2013 [31]	-	-	28.6	71.4	15.0
Ekstrand et al., 2004 [51]	80.0	20.0	-	-	-
Ekstrand et al., 2011b [13]:					
UEFA-UCL	-	-	-	-	13
SWE	-	-	-	-	22
ART	-	-	-	-	20
Hägglund et al., 2005a [33]:					
Denmark	61.0	39.0	-	-	30.0
Sweden	62.0	38.0	-	-	24.0
Hägglund et al., 2006 [26]:					
Season 2001	63.1 *	36.9 *	-	-	22.1 *
Season 2002	60.5 *	39.5 *	-	-	18.2 *
Hägglund et al., 2007 [28]					
EURO 2004	80	20	53	47	-
U-19 2005	94	6	59	41	-
Hägglund et al., 2009 [19]:					
Men Under-21, 2006	77.0	23.0	65.0	35.0	9.0
Men Under-21, 2007	68.0	32.0	59.0	41.0	4.0
Men EURO, 2008	73.0	27.0	73.0	27.0	7.0
Hägglund et al., 2016 [48];					
Top-Level	-	-	-	-	16.6
Elite	-	-	-	-	26.0
Hawkins et al., 1999 [14]	-	-	41.0	59.0	22.0
Jones et al., 2019 [37]	60.0	40.0	-	-	16.9
Kordi et al., 2011 [24]	89.0	11.0	59.2	40.8	-
Lee et al., 2014 [43]	84.0	16.0	45.0	55.0	21.0
Mallo et al., 2011 [30];	60.0	40.0	-	-	9.0
Under Elite					
Martins et al., 2022 [52]:					
Season 2019/2020	30.8	69.2	-	-	11.5
Season 2020/2021	50.0	50.0	-	-	11.8
Season 2021/2022	58.4	33.3 **	-	-	12.5
Murphy et al., 2012 [44]	-	-	32.2	67.8	23.0
Noya et al., 2014b [45]	34.6	65.4	45.0	55.0	12.5
Noya et al., 2014a [46]	34.3	65.7	-	-	11.2
Reis et al., 2015 [29]	21.4	78.6	20.0	80.0	7.1
Roe et al., 2018 [6]	81.7	18.3	36.8	63.2	22.0
Shalaj et al., 2016 [9]	70.96	29.04	-	-	-
Stubbe et al., 2015 [5]	68.5	31.5	39.3	60.7	-
Waldén et al., 2005a [47]	73.0	27.0	-	-	15.0
Walden et al., 2005b [18]	63.5	36.5	-	-	22.0
Waldén et al., 2007 [34]:					
EURO 2004	80.0	20.0	53.0	47.0	-
U-19 EC 2005	94.0	6.0	41.0	59.0	-
Mean value ± SD	64.16 ± 15.34	35.60 ± 15.22 **	50.70 ± 11.56	49.30 ± 11.56	15.26 ± 5.81

* Calculated values; ** the difference of 8.3% in other causes (Martins et al. [52], Season 2021/2022). a. Swedish Super League (SWE); b. UEFA Champions League (UCL); ART—Artificial Turf Field; 12%—slight injuries, Hägglund et al. [19].

**Table 4:** Mechanism of injury in amateur football players.

Study	Traumatic Injuries, %	Overuse Injuries, %	Contact Injuries, %	Non-Contact Injuries, %	Recurrent Injuries, %
Kekelekis et al., 2023 [22]	-	-	15.0	85.0	11.7 *
Kordi et al., 2011 [24]:					
ATF	89.0	11.0	59.2	40.8	-
DF	91.0	9.0	51.4	48.6	-
Hägglund et al., 2016; U-19 [48]	-	-	-	-	35.1
Hammes et al., 2015 [32]:					
Intervention Group (INT) **	58.8 *	39.2 *	33.0	67.0	37.0
Group Control (CON)	59.0	41.0	38.0	62.0	24.0
Herrero et al., 2014 [49]:					
<30 years	-	-	25.4	25.5	2.5
>30 years	-	-	74.6	74.5	2.5
Nogueira et al., 2017 [50] U-17 + U-19	76.6	23.4	-	-	-
Sousa et al., 2013 [23]	79.0	21.0	49.0	51.0	10.0
Van Beijsterveldt et al., 2012 [53]:					
Intervention Group (INT)	78.9	21.1	68.0	32.0	13.0
Control Group	82.7	17.3	-	-	14.1
Mean values ± SD	76.88 ± 9.05	22.86 ± 8.74	45.96 ± 16.1	55.04 ± 16.1	16.66 ± 10.25

** Hammes et al. (INT) [32]—Traumatic injuries + overuse missing 2%; * Calculated Values.

**Table 5:** Severity of injuries—Professional football.

Study	Injuries of Severity			
	Minimal	Mild	Moderate	Severe
	(1–3 days), %	(4–7 days), %	(8–28 days), %	(>28 days), %
Aus der Fünten et al., 2014 [38]:				
2008–2009	28.48	29.14	29.14	13.24
2009–2010	30.2	18.8	33.6	17.4
Aus der Fünten et al., 2023 [39]	32.7	20.6	28.1	18.6
Bayne et al., 2018 [40]	21.2 *	12.1 *	36.4 *	30.3 *
Dupont et al., 2010 [27]:				
Group G1	37.9 *	27.6 *	29.3 *	5.2 *
Group G2	24.3 *	30.84 *	30.84 *	14.2
Eirale et al., 2010 [42]	33.3 *	30.8 *	29.5 *	6.4 *
Ekstrand et al., 2011a [7]	21.7 *	26.0 *	36.8 *	15.5 *
Ekstrand et al., 2011b [13]:				
Sweden. Total	15.0	27.0	47.0	11.0
UEFA-UCL	12.0	24.0	51.0	13.0
SWE	24.0	33.0	35.0	8.0
ART	15.0	32.0	47.0	6.0
Hägglund et al., 2005a [33]				
Denmark	43.0	24.0	21.0	12.0
Sweden	36.0	24.0	31.0	9.0
Hägglund et al., 2006 [26]				
Season 2001	30.1 *	27.1 *	32.8 *	10.0 *
Season 2002	36.2 *	28.1 *	24.7 *	11.0 *
Hägglund et al., 2007 [28]: Season 2005	36.0	29.0	26.0	9.0
Hägglund et al., 2009 [19]:				
European Championship (EC) 2006	54.54 *	9.09 *	13.64 *	22.73 *
European Championship (EC) 2007 **	32.0	16.0	32.0	8.0
European Championship (EC) 2008	39.3	16.1	21.4	23.2
Hawkins et al., 1999 [14]	16.4 *	36.0 *	37.0 *	10.6 *
Jones el al., 2019 [37] **	35.94	4.65	44.19	15.01
Lee et al., 2014 [43]	60.5 *	14.2 *	18.2 *	7.1 *
Mallo et al., 2011 [30]	51.1 *	22.4 *	22.7 *	3.8 *
Martins et al., 2022 [52].				
Season 2019/2020	7.8	19.3	69.3	3.6
Season 2020/2021	11.8 *	20.5	50.0	17.7 *
Season 2021/2022	16.7	29.1	37.5	16.7
Morgan et al., 2001 [16]	0	60.0	26.0	14.0
Murphy et al., 2012 [44]	0	13.2	45.2	41.6
Noya et al., 2014b [45]	40.1	23.8	27.7	8.4
Noya et al., 2014a [46]	35.7	26.8	29.2	8.3
Reis et al., 2015 [29]	22.9	21.4	40.0	15.7
Roe et al., 2018 [6]	0	27.0	49.8	23.2
Shalaj et al., 2016 [9]	16.2	34.2	39.7	9.90
Stubbe et al., 2015 [5] **	17.5	31.8	34.3	15.4
Waldén et al., 2005a [47]	27.7 *	28.3 *	29.3 *	14.7 *
Waldén et al., 2005b [18]	32.4 *	27.4 *	30.8 *	9.4 *
Waldén et al., 2007 [34]				
Euro 2004	47.0	13.0	13.0	6.0
U-19 2005	47.0	18.0	29.0	27.0
Mean values ± SD	27.94 ± 11.93	24.52 ± 6.71	33.57 ± 8.37	13.32 ± 5.59

* Calculated values; ** Unknown causes: 1%, Stubbe et al. [5]; SD—standard deviation; Jones et al. [37]: 0.21% unspecified causes. a. Swedish Super League (SWE); b. UEFA Champions League (UCL); ART—Artificial Turf Field; 12%—slight injuries, Hägglund et al. [19].

**Table 6:** Severity of injuries—Amateur Football.

Study	Severity of Injuries			
	Minimal + Slight	Mild	Moderate	Severe
	(1–3 days), %	(4–7 days), %	(8–28 days), %	(>28 days), %
Hammes et al., 2015 [32]:				
InterventionGroup (INT)	0	13.7 *	64.7 *	21.6 *
ControlGroup (CG)	0	19.0	35.0	46.0
Hawkins et al., 1999 [14]	16.4	36.0	37.0	10.6
Kekelikis et al., 2023 [22]	32.0	40.8	23.3	3.9 *
Nogueira et al., 2017 [50]. U-17 + U-19	13.31 *	22.98 *	43.15 *	20.56 *
Sousa et al., 2013 [23]	9.0	29.0	40.0	22.0
Van Beijsterveldt et al., 2012 [53]:				
InterventionGroup	5.9	18.5	46.3	28.8
ControlGroup **	5.1	21.5	41.6	29.9
Mean Values ± SD	10.21 ± 7.77	25.19 ± 7.56	41.38 ± 7.56	22.92 ± 9.0

SD—standard deviation; * Calculated values; ** Van Beijsterveldt et al. [53]: Intervention—0.5% slight (0 days), 1.9% Control—0.5% slight (0 days) and 1.4% total loss of ability to play football.

## Data Availability

Data are contained within the article.

## Supplementary Materials

The following supporting information can be downloaded at: https://www.mdpi.com/article/10.3390/jcm12196293/s1, Table S1: PRISMA checklist; Table S2: Definitions used to include studies in systematic review; Table S3: Characteristics of the studies included in the review: General descriptors of study; Description of the study population (n = 48); Table S4: Characteristics of the studies included in the review: Epidemiological descriptorsMethodological quality; Table S5: Analysis of the selected studies’ methodological quality—STROBE (n = 46); Table S6: Risk of bias assessment of the studies (Newcastle-Ottawa scale; n = 46); Figure S1: PRISMA flow chart.
