# Supplementary material for: Epidemiology of Injuries in Professional and Amateur Football Men (Part II)
Source: J Clin Med. 2023 Sep 29;12(19):6293. doi: 10.3390/jcm12196293 (PMC10573283; doi:10.3390/jcm12196293)
Supplement: Supplementary file 1 [file jcm-12-06293-s001.zip › jcm-2566704-supplementary.pdf]

Additional material
Table S1 PRISMA checklist

Section/topic		Checklist item	Reported on page #
**TITLE**			
Title	1	Identify the report as a systematic review, meta-analysis, or both.	
**ABSTRACT**			
Structured summary	2	Provide a structured summary including, as applicable: background; objectives; data sources; study eligibility criteria, participants, and interventions; study appraisal and synthesis methods; results; limitations; conclusions and implications of key findings; systematic review registration number.	
**INTRODUCTION**			
Rationale	3	Describe the rationale for the review in the context of what is already known.	
Objectives	4	Provide an explicit statement of questions being addressed with reference to participants, interventions, comparisons, outcomes, and study design (PICOS).	
**METHODS**			
Protocol and registration	5	Indicate if a review protocol exists, if and where it can be accessed (e.g., Web address), and, if available, provide registration information including registration number.	
Eligibility criteria	6	Specify study characteristics (e.g., PICOS, length of follow-up) and report characteristics (e.g., years considered, language, publication status) used as criteria for eligibility, giving rationale.	
Information sources	7	Describe all information sources (e.g., databases with dates of coverage, contact with study authors to identify additional studies) in the search and date last searched.	
Search	8	Present full electronic search strategy for at least one database, including any limits used, such that it could be repeated.	
Study selection	9	State the process for selecting studies (i.e., screening, eligibility, included in systematic review, and, if applicable, included in the meta-analysis).	
Data collection process	10	Describe method of data extraction from reports (e.g., piloted forms, independently, in duplicate) and any processes for obtaining and confirming data from investigators.	
Data items	11	List and define all variables for which data were sought (e.g., PICOS, funding sources) and any assumptions and simplifications made.	
Risk of bias in individual studies	12	Describe methods used for assessing risk of bias of individual studies (including specification of whether this was done at the study or outcome level), and how this information is to be used in any data synthesis.	
Summary measures	13	State the principal summary measures (e.g., risk ratio, difference in means).	
Synthesis of results	14	Describe the methods of handling data and combining results of studies, if done, including measures of consistency (e.g., *I*2) for each meta-analysis.	
Risk of bias across studies	15	Specify any assessment of risk of bias that may affect the cumulative evidence (e.g., publication bias, selective reporting within studies).	

Additional analyses	16	Describe methods of additional analyses (e.g., sensitivity or subgroup analyses, meta-regression), if done, indicating which were pre-specified.
**RESULTS**		
Study selection	17	Give numbers of studies screened, assessed for eligibility, and included in the review, with reasons for exclusions at each stage, ideally with a flow diagram.
Study characteristics	18	For each study, present characteristics for which data were extracted (e.g., study size, PICOS, follow-up period) and provide the citations.
Risk of bias within studies	19	Present data on risk of bias of each study and, if available, any outcome level assessment (see item 12).
Results of individual studies	20	For all outcomes considered (benefits or harms), present, for each study: (a) simple summary data for each intervention group (b) effect estimates and confidence intervals, ideally with a forest plot.
Synthesis of results	21	Present results of each meta-analysis done, including confidence intervals and measures of consistency.
Risk of bias across studies	22	Present results of any assessment of risk of bias across studies (see Item 15).
Additional analysis	23	Give results of additional analyses, if done (e.g., sensitivity or subgroup analyses, meta-regression [see Item 16]).
**DISCUSSION**		
Summary of evidence	24	Summarize the main findings including the strength of evidence for each main outcome; consider their relevance to key groups (e.g., healthcare providers, users, and policy makers).
Limitations	25	Discuss limitations at study and outcome level (e.g., risk of bias), and at review-level (e.g., incomplete retrieval of identified research, reporting bias).
Conclusions	26	Provide a general interpretation of the results in the context of other evidence, and implications for future research.
**FUNDING**		
Funding	27	Describe sources of funding for the systematic review and other support (e.g., supply of data); role of funders for the systematic review.

From: Moher et al. (2009) [33]

Table S2 Definitions used to include studies in systematic review

Term	Definition
Injury	Any physical complaint sustained by a player that results from a football match or football training, irrespective of the need for medical attention or time loss from football activities
Time loss injury	Injury that results in a player being unable to take a full part in future Football training or match play.
Recurrent injury	Injury of the same type and at the same site as an index injury and which occurs after a player's return to full participation from the index injury.
Injury severity	The number of days that have elapsed from the date of injury to the date of the player's return to full participation in team training, and availability for match selection. Injuries are grouped as:<br>Slight / Minimal Absence (1-3 days);<br>Minor / Mild Absence (4-7 days);<br>Moderate Absence (8-28 days);<br>Major / Severe Absence (>28 days).
Match exposure	Play between teams from different clubs.

Training exposure	Team-based and individual physical activities under the control or guidance of the team's coaching or fitness staff that are aimed at maintaining or improving players' football skills or physical condition.
Traumatic injury:	Injury with sudden onset and known cause.
Sprain	Acute distraction injury of ligaments or joint capsules
Strain	Acute distraction injury of muscles and tendons
Contusion	Tissue bruise without concomitant injuries classified
Fracture	Traumatic break of bone
Dislocation	Partial or complete displacement of the bony parts of a joint
Other	Injuries not classified elsewhere (wound, concussion).
Overuse	A pain syndrome of the musculoskeletal system with.
Contact Injury	An injury caused by external influence (any contact with another player or object).
Non-contact injury	An injury that occurred without external influence.
Location of injury	• Head and neck (Head/face; Neck/cervical spine);<br>• Upper limbs (Shoulder/clavicle; Arm; Elbow; Forearm; Wrist; Hand/finger/thumb);<br>• Trunk (Sternum/ribs/upper back; Abdomen; Lower back/pelvis/sacrum);<br>• Lower Limbs (Hip/Groin; Thigh; Knee; Lower Leg/Achilles; Ankle; Foot/Toe).
Injury incidence	Number of injuries per 1000 player hours = [(Σ injuries/Σ exposure hours) ×1000].
Confidence intervals	95% confidence intervals = [Incidence x/e$^{(1.96 \times \sqrt{(1/\text{injuries})})}$]
Professional football players	Players who belong to teams engaged in professional national Football leagues. Frequently, these leagues are the country's two highest divisions [first league, second league].
Level of play	
International	UEFA defines international football as a "match between two national teams composed of the best eligible players."
Elite	The highest national football league.
Amateur	Only league below the highest national football league.

Source: Lindenfeld, et al. (1988) [36]; Hägglund et al. (2005)[16]; Fuller et al. (2006) [35]; Lopez et al. (2020) [20]

Table S3 Characteristics of the studies included in the review:General descriptors of study; Description of the study population (n=48)

Study	Article Title	Region	Study Design	Status	Number players	Mean ± SD age, years	Duration study
Arnason et al., 2005 [35]	No Effect of a Video-Based Awareness Program on the Rate of Football Injuries	Iceland	Prospective cohort study	Professional Elite and Premier Division	271	24.0 (16-38)	May-September 2000
Aus der Fünten et al., 2014 [38]	Injury Characteristics in the German Professional Male Football Leagues After a Shortened Winter Break	Germany	Prospective cohort study	Professional football leagues	184<br>188	25.2±4.1<br>25.2±4.3	2 Seasons:<br>2008-2009<br>2009-2010
Aus der Fünten et al., 2023 [23]	Epidemiology of Football Injuries of the German Bundesliga: A Media-Based, Prospective Analysis over 7 Consecutive Seasons	Germany	Prospective cohort study	Professional Premier League	650	25±4.0	7 Seasons: 2014-2015 to 2020-2021

Bayne et al., 2018 [39]	Incidence of injury and illness in South African professional male football players: a prospective cohort study	South Africa	Prospective cohort study	Professional	56	-	10 Months 2015-2016
5. Brito et al., 2012 [25]	Injuries in Portuguese Youth Football Players During Training and Match Play	Portugal	Descriptive epidemiological study	Young amateur footballers U-19	674<br>161	Different ages<br>17-18 years old	1 Season August 2008-June 2009
Dvorak et al., 2011 [40]	Injuries and illnesses of football players during the 2010 FIFA World Cup	FIFA World Cup 2010		Elite Professional	533		Year: 2010
Dupont et al., 2010; France [41]	Effect of 2 Football Matches in a Week on Physical Performance and Injury Rate	France	Prospective cohort study	Professional Level: Top UEFA	32	25.6±3.8	2 Seasons 2007-2008; 2008-2009
Eirale et al., 2010 [42]	Injury epidemiology in a national football team of the Middle East	Qatar	Prospective epidemiological study	Professional football	36	23.8	17 Months June 2007-October 2008
Eirale et al., 2013[43]	Epidemiology of football injuries in Asia: A prospective study in Qatar	Qatar	Prospective cohort study	Professional First division	230	28.4±4.4	August 2008-April 2009
Ekstrand et al., 1990 [44]	The incidence of ankle sprains in football	Sweden	Prospective cohort study	Professional Different divisions	315	-	1 Year
Ekstrand et al., 2004a [45]	A congested football calendar and the wellbeing of players: correlation between match exposure of European footballers before the World Cup 2002 and their injuries and performances during that World Cup	Europe (World Cup and Non-World Cup)	Prospective cohort study	Professional	266	26	10 Months; July 2001-May 2002
Ekstrand et al., 2004b [46]	Risk for injury when playing in a national football team	Sweden NT; World Cup 1994, European Championship 1992	Prospective cohort study	Professional	-	-	6 years: 1991-1997
Ekstrandet al., 2011a [47]	Injury incidence and injury patterns in	Europe Sweden	Prospective cohort study	Professional UCL*	2226	25.7±4.5	2 Seasons each: 2001-2008

	professional football - the UEFA injury study			SWE*			
Ekstrandet al., 2011b [14]	Epidemiology of muscle injuries in professional football (football)	Sweden: UCL; SWE; ART	Prospective cohort study	Professional footballers	2299	25.3±4.6	9 Seasons: 2001-2009
Fischer et al., 2017 [48]	Injuries in amateur football. Collecting data for injury prevention	Austria	Prospective cohort study	Amateur footballers, level 3-4	127	21.9±4.5	1 Season
Gebert et al., 2018 [29]	Changes in injury incidences and causes in Swiss amateur football between the years 2004 and 2015	Switzerland	Retrospective study	Amateur footballers	-	Different ages	3 Years: 2004, 2008, 2015
Hägglande et al., 2005a[16]	Injury incidence and distribution in elite football-a prospective study of the Danish and the Swedish top divisions	Denmark Sweden	Prospective cohort study	Elite Professional	188<br>310	26±4<br>25±5	January-June 2001<br>January November
Hägglund et al., 2006[17]	Previous injury as a risk factor for injury in elite football: a prospective study over two consecutive season.	Sweden	Prospective cohort study	Elite Professional	263;<br>262	25 ±5<br>25±5	January. 2001-November 2002 (2 Seasons)
Hägglund et al., 2007a49	Epidemiology and prevention of football injuries	Sweden<br>Sweden<br>Denmark<br>Sweden	Prospective study	Elite Professional, Top division	118<br>310<br>188<br>239	25±3<br>25 ±5<br>26 ±4<br>25 ±5	Ian-Oct. 1982<br>Ian-Oct 2001<br>Ian-Oct. 2001<br>Ian-Oct. 2001
Hägglund et al., 2009 [50]	UEFA injury study - An injury audit of European Championships 2006 to 2008	European Championship	UEFA Study	Professional footballers;<br>U-21	176 (2006)<br>182 (2007)<br>367 (2008)	27.2±4.0<br>21.6 ±1.2<br>21.3±1.3	Championships 2006 to 2008
Hägglund et al., 2013[51]	Injuries affect team performance negatively in professional football: an 11-year follow-up of the UEFA Champions League injury study	EURO League match UCL or EL match	Prospective cohort study	Professional (9 European countries)	155 teams-seasons	-	11 Seasons 2001-1012
Hägglund et al., 2016 [19]	Injury recurrence is lower at the highest professional football level than at national and amateur levels: does sports medicine and sports physiotherapy deliver ?	Europe Sweden	Prospective cohort study	Professional Elite Level-Top Amateur	Players<br>2014<br>6956<br>241	25.2±4.8<br>25.4±4.6<br>24.0±5.3	2001-1015<br>14 Seasons
Hammes et al., 2014 [52]	Injury prevention in male veteran football players – a randomized	Germany	Prospective cohort study	Veteran amateur football	265	45±8	2011-2012

	controlled trial using "FIFA 11+".			players, Level 1-3			
Hawking et al.,1999 [53]	A prospective epidemiological study of injuries in four English professional football clubs	England	4 English leagues, competition	Professional	108	-	1994-1997 November-May, 407 weeks
Herrero et al., 2014 [54]	Injuries among Spanish male amateur football players: a retrospective population study	Spain	Retrospective epidemiological study	Amateur footballers	134.570	18-55	2010-2011
Jones et al., 2019[55]	Epidemiology of injury in English Professional Football players: A cohort study	England	Prospective cohort study	Professional English Football League; clubs	243	24.3±4.21	1 Season July 2015-May 2016
Kekelekis et al., 2023 [30]	Epidemiology of Injuries in Amateur Male Football Players: A Prospective One-Year Study	Greece	Prospective cohort study	Amateur footballers	152	21.32±7.42	1 Season 2018-2019 (26 Weeks)
Kordi et al., 2011[56]: Earth Field (DF) Artificial Turf (ATF)	Comparison of the incidence, nature and cause of injuries sustained on dirt field and artificial turf field by amateur football players	Iran	Prospective cohort study	Amateur footballers	DF 252<br>ATF 216	27.0 (18-43)<br>28 (17-40)	13 Weeks
Lee et al., 2014 [57]	A prospective epidemiological study of injury incidence and injury patterns in a Hong Kong male professional football league during the competitive season.	Asia Hon Kong	Prospective study	Professional	152	25.0±4.3	1 Season: September. 2010- May 2011
Mallo et al., 2011 [58]	Injury Incidence in a Spanish Sub-Elite Professional Football Team: A Prospective Study During Four Consecutive Seasons	Spain	Prospective cohort study	Professional Sub-elite teams; Division II	88	24.8±3.5	4 Seasons: 2003-2004; 2004-2005; 2005-2006 2006-2007
Martins et al., 2022 [59]	Sports Injuries of a Portuguese Professional Football Team during Three Consecutive Seasons	Portugal	Prospective operational study	Professional Premier league	71	25.7±3.4	3 Seasons: 2019-2020; 2020/2021; 2021-2022
Morgan et al., 2001[60]	An examination of injuries in Major League Football: the inaugural season	SUA	Prospective cohort study	Professional High level	237	27.0	7 Months (1 Season)

Murphy et al., 2012 [61]	Incidence of Injury in Gaelic Football: A 4-Year Prospective Study	Ireland	Descriptive epidemiological study	Professional Elite teams in Wales	851	18-36	4 Seasons: 2007-2010
Nogueira et al., 2017[28]	Injuries in Portuguese Amateur Youth Football Players: A Six Month Prospective Descriptive Study	Portugal	Descriptive observational study	Amateur football players U19	239	18-19	November 2015- April 2016
Noya Salces et al., 2014b [62]	An examination of injuries in Spanish professional football League	Spain	Prospective cohort study	Professional Second division	301	26,4±4,0	1 Season: 2008-2009
Noya Salces et al., 2014a [63]	Epidemiology of injuries in First Division Spanish football	Spain	Prospective cohort study	Professional Premier League	427	26.8±4.1	6 July 2008-23 August 2009
Parry and Drust et al., 2006[64]	Is injury the major cause of elite football players being unavailable to train and play during the competitive season?	England	Descriptive epidemiological study	Professional Division I	55	24.0±5	Competitive season 2003-2004 2004-2005
Reis et al., 2015 [65]	Sports injuries profile of a first division Brazilian football team: a descriptive cohort study	Brazil	Prospective cohort study	Professional First division	48	25.2±4.5	1 Season
Roe et al., 2018 [66]	Time to get our four priorities right: an 8-year prospective investigation of 1326 player-seasons to identify the frequency, nature, and burden of time-loss injuries in elite Gaelic football	Ireland	Prospective study	Professional	1326 player - seasons	Different ages (18-40 years)	8 Years, 2008-2016
Shalaj et al., 2016[67]	Injuries in professional male football players in Kosovo: a descriptive epidemiological study	Kosovo	Prospective cohort study	Professional footballers	143	23.2±4.1	1 Season, 2013-2014
Sousa et al., 2012 [26]	Injuries in amateur football players on artificial turf: A one-season prospective study	Portugal	Prospective cohort study	Amateur footballers	231	24.7 (18-38)	1 Season August. 2010-Mai 2011
Stubbe et al., 2015 [68]	Injuries in Professional Male Football Players in the Netherlands: A Prospective Cohort Study	Netherlands	Prospective epidemiologic cohort study	Professional First league	217	24.6±4.3	31 July 2009-2 May 2010
Waldén et al., 2005a [69]	UEFA Champions League study: a prospective study of	Europe	Prospective cohort study	Professional High level	266	26±4	9 Months(July

	injuries in professional football during the 2001-2002 season						2001-May 2002)
Waldén et al., 2005b[4]	Injuries in Swedish elite football: a prospective study on injury definitions, risk for injury and injury pattern during 2001	Sweden	Prospective cohort study	Professional Top level	310	25 (17-28)	2001 (Ianuar-Octomber)
Waldén et al., 2007 [70[	Football injuries during European Championships 2004–2005	Europe	Prospective study	Professional<br>EURO 2004<br>WOCO 2005<br>U-19 2005	672:<br>368<br>160<br>144	-	12 June-7 July; 2004;<br>5 June-19 July 2005;<br>18 July-29 July 2005
Van Beijsterveldt et al., 2012 [27]	Effectiveness of an injury prevention programme for adult male amateur football players: A cluster-randomized controlled trial	Netherlands	Cluster randomized controlled trial	Amateur footballers	223 (INT)<br>233 Control)	24.4±4.1<br>25.1±4.3	2009-2010

*UCL-UEFA Champions League; SWE-Swedish First League; ART-Artificial Turf Field; - non provide

Table S4 Characteristics of the studies included in the review: Epidemiological descriptorsMethodological quality

Reference Country / Tournament	Study duration	No teams Players	Exposure (Hours)			Injuries			Incidence			Strobe Quality	NOS Methodological quality
			Overall	Training	Match	Overall	Training	Match	Overall	Training	Match		
Arnason et al., 2005 [37]	May 1999-September 1999	15<br>271	28,927	23,613	5,314	190	136	54	6.60	1.90	26.0	7	7
Aus der Fünten et al., 2014 [38]	Season: 2008-2009 2009-2010	14<br>372	48,285.6	42,817.2	5,468.4	300	151	149	6.2*	3.5*	27.2*	10	8
Aus der Fünten et al., 2023 [23]	7 Seasons: 2014/2015-2020/2021	25<br>650	1,220,223.5	1,111,03	109,193.5	6,653	3,821	2,832	5.5	3.4	25.9	8	8
Bayne et al., 2018 [39]	10 Months 2015-2016	2<br>56	20,361	19,272	1,089	33	15	18	1.6	0.8	16.5	8	6

5. Brito et al., 2012 [25]	August 2008- Iuly 2009	14<br>674<br>U-19	23.122<br>6682	21.401<br>6147	1.721<br>1721	199<br>76	139<br>-	60<br>-	8.6<br>11.3	6.5<br>-	54.9<br>-	7	6
Dvorak et al., 2011 [40]	1 Year 2010	32<br>553	-	-	-	229	104	125	9.20	7.9	61.1	7	5
Dupont et al., 2010; France (G1, G2) [41]	2 Seasons: 2007-2008 2008-2009	1<br>32	18,495	16,339	2,156	165	105	60	8.9	3.7	48.7	8	8
Eirale et al., 2010 [42]	June 2007- Octomb er 2008, 17 Months	/36	10,043	9,482	561	78	41	37	7.8	4.3	66.0	9	7
Eirale et al., 2013[43]	1 Season: August 2009- April 2009	10<br>239	36,020	30,227	5,793	217	133	84	6.02*	4.4	14.5	7	7
Ekstrand et al., 1990 [44]	1 Year	21<br>315	30,554<br>31,718	23,241<br>24,499	7,313<br>7,219	261<br>288	107<br>-	159<br>-	8.6<br>8.5	4.6<br>5.1	21.8<br>18.7	9	6
Ekstrand et al., 2004a [45]	6 Years: 1991-1997	/73	7,245	6,235	1,010	71	40	31	10.0	6.5	30.3	6	5
Ekstrand et al., 2004b [46]	July 2001- May 2002	11<br>266	70,000	58,000	12,000	-<br>-	-<br>-	-<br>-	7,9<br>9,5	3,2<br>5,5	26,7<br>30,3	7	5
Ekstrandet al., 2011a [47]	7 Seasons: 2001-2008	88<br>2226	566,000	475,000	91,000	4,483	1,937	2,546	8.0	4.1	27.7	8	7
Ekstrandet al., 2011b [14]	2001-2009	51<br>2.299	1,175,000	998,000	177,000	2,908	1,541	1,367	2.48	1.37	8.70	8	7
Fischer et al., 2017 [48]	1 Season	1<br>127	15,309	12,195	3,114	62	25	37	4.05	2,05	11,06	-	-
Gebert et al., 2018 [29]	2004<br>2008<br>2015	-<br>-<br>-	-<br>-<br>-	182,961	-<br>-<br>-	1,601	525	1,076	-<br>-<br>-	2.4<br>2.2<br>2.9	15.1<br>13.3<br>16.5	6	5
Häggland et al., 2005a[16]	January 2001- June 2001	8<br>188<br>14<br>310	27,321<br>59,469	23,095<br>52,910	4,226<br>6,559	349<br>481	271<br>313	124<br>168	14.4<br>8.2	11.8<br>6.0	28.2<br>26.2	8	7
Hägglund et al., 2006[17]	2 Seasons: 2001 and 2002	525	155,867	135,822	20,045	1,189	701	488	7.6	5.16	24.2	8	8

Hägglund et al., 2007a49	Years:<br>1982,<br>2001,<br>2002,<br>2005	48<br>933	-	-	-	715<br>588<br>548			8.3<br>7.8<br>7.6<br>7.7	4.6<br>5.2<br>5.3<br>4.7	20.6<br>25.9<br>22.7<br>28.1	8	7
Hägglund et al., 2009 [50]	Year:<br>2006	8<br>176	1,589	1,076	513	22	5	17	13.8	4.6	33.1	8	7
	Year200<br>7	8<br>182	2,321	1,774	548	25	6	19	10.8	3.4	34.7		
	Year:200<br>8	16<br>367	5,368	4,310	1,058	56	12	44	10.4	2.8	41.6		
Hägglund et al., 2013[51]	11<br>Seasons<br>2001-<br>1012	155<br>Teams<br>-<br>Seaso<br>ns	1,026,11<br>4	-	-	7,792	3,395	4,39<br>7	7.7	4.0	36.6	8	5
Hägglund et al., 2016 [19]	2001-<br>2015	6,956<br>2,014<br>241	-<br>-<br>-	-<br>-<br>-	-<br>-<br>-	9,757<br>3,179<br>114	-<br>-<br>-	-<br>-<br>-	7.2<br>7.4<br>5.2	3.8<br>4.9<br>2.7	25.3<br>23.5<br>12.3	7	5
Hammes et al., 2014 [52]	2011-<br>2012;<br>9<br>Months	18<br>265	7,109	4,798	2,311	88	31	57	12.3<br>7*	6.46	24.6<br>6*	7	6
Hawking et al.,1999 [53]	1994-<br>1997<br>407<br>Weeks	/108				578	187	391	8.5	27.7	3.5	8	7
Herrero et al., 2014 [54]	1 Season<br>2010-<br>2011	134.57<br>0<br>Player<br>s	-	-	-	15,243	10,256	4,98<br>7	-	0.49	1.15	7	7
Jones et al., 2019[55]	1 Season<br>2015-<br>2016	10<br>243	56,075	46,351	9,724	473	-	-	9.11	6.84	24.2<br>9	9	6
Kekelekis et al., 2023 [30]	1 Season<br>2018-<br>2019	11<br>152	18,558	15,909	2,648	103	48	55	5.5	3.46	18.1<br>2	9	8
Kordi et al., 2011[56]:<br>Earth Field (DF)	13<br>Weeks	14<br>252	3275<br>1897	-	-	97<br>70	-	-	-	-	29.6<br>36.9	6	5
Artificial Turf (ATF)		12<br>216	1,378			27					19.5		
Lee et al., 2014 [57]	1 Season<br>2010-<br>2011	10<br>152	39,824	36,936	2,888	296	-	-	7.4	3.4	61.6	8	6
Mallo et al., 2011 [58]	4<br>Seasons,	1<br>88	28,694	24,509	4,185	313	129	184	10.,9	5.2	44.1	8	7

	2003-2007												
Martins et al., 2022 [59]	2019-2020<br>2020-2021<br>2021-2022	3<br>104	7,821.6<br>7,794.3<br>8,430.0	7,179.5<br>7,154.8<br>7,780.0	642.1<br>639.6<br>650.0	26<br>34<br>24	17<br>20<br>17	9<br>14<br>7	3.3<br>4.4<br>2.8	2.4<br>2.8<br>2.2	14.0<br>21.9<br>10.8	8	6
Morgan et al., 2001[60]	1 Season 7 Months	10<br>237	-	-	-	256	-	-	6.2	2.9	35.3	7	5-
Murphy et al., 2012 [61]	4 Seasons 2007-2010	/851	106,890	97,950	8,940	1014	397	553	8.89*	4.05	61.86	7	7
Nogueira et al., 2017[28]	November 2015-April 2016	21<br>529	66,062	53,159.5	8,902.5	248	119	129	3.87	2.06	14.22	8	7
Noya Salces et al., 2014b [62]	2008-2009	11<br>301	161,602.7	153,567.2	8,035,5	891	579	312	5,51	3,77	38,83	9	8
Noya Salces et al., 2014a [63]	July 2008-May 2009	16<br>427	229,443	216,705	12,738	1,293	769	524	5.65	3.55	43.53	9	8
Parry and Drust et al., 2006[64]	2003-2005	/55	13,346	10,742	2,604	83*	19*	64*	6,2	1,8	24,6	7	5
Reis et al., 2015 [65]	1 Season (334 days)	1<br>*48*	13,040*	12,083	957*	70	29	41	5.37	2.40	42.84	8	7
Roe et al., 2018 [66]	8 Years	-	177,854	159,866	17,988	1512*	616	896	8,50	3,85	49,8	8	7
Shalaj et al., 2016[67]	2013-2014	11<br>143	36,833	31,998	4,834	272	101	171	7.38	3.16	35.37	9	7
Sousa et al., 2012 [26]	August 2010-May 2011	11<br>231	43,872	38,554	5,318	213	92	121	4.86	2.39	22.75	8	7
Stubbe et al., 2015 [68]	31 July 2009-2 May 2010	8<br>217	46,194	41,012	5,182	286	170	116	6.2	2.8	32.8	11	7
Waldén et al., 2005a [69]	9 Months July 2001-May 2002	11<br>266	69,707	58,149	11,558	658	298	360	9.4	5.8	30.5	8	6
Waldén et al., 2005b[4]	Ianuar-Octomber 2001	14<br>310	93,353	81,801	11,552	715	421	294	7.66	5.15	25.45	8	7
Waldén et al., 2007 [70[	European	32<br>672	7,957:	5,907	2,050	80	12	68	10.1	2.03	33.2	8	7

Van Beijsterveldt et al., 2012 [27]	Championships 1 Season 2009-2010	23 456	44,252	31,518	12,734	424	-	-	9.6	3.4	21.9	8	8

Table S5 Analysis of the selected studies' methodological quality - STROBE (n = 46)

Study: Author, Year	1	2	3	4	5	6	7	8	9	10	11	Scoring
Arnason et al., 2005 [37]	+	-	-	-	-	+	+	+	+	-	+	7
Aus der Fünten et al., 2014 [38]	+	+	+	+	+	+	+	+	+	-	+	10
Aus der Fünten et al., 2023 [23]	+	+	-	-	+	+	+	+	+	-	+	8
Bayne et al., 2018 [39]	+	+	+	-	-	+	+	+	+	-	+	8
Brito et al., 2012 [25]	+	-	-	-	+	+	+	+	-	-	+	7
Dvorak et al., 2011 [40]	+	-	-	-	+	-	+	+	+	-	+	7
Dupont et al., 2010; France [41]	+	+	+	-	-	+	+	+	+	-	+	8
Eirale et al., 2010 [42]	+	+	+	-	+	+	+	+	+	-	+	9
Eirale et al., 2013[43]	+	+	+	-	+	-	+	+	-	-	+	7
Ekstrand et al., 1990 [44]	+	-	-	-	-	-	+	+	+	-	+	5
Ekstrand et al., 2004a [45]	+	+	-	-	-	+	+	-	+	-	+	6
Ekstrand et al., 2004b [46]	+	+	-	-	+	+	+	+	-	-	+	7
Ekstrandet al., 2011a [47]	+	+	+	-	-	+	+	+	+	-	+	8
Ekstrandet al., 2011b [14]	+	+	+	-	-	+	+	+	+	-	+	8
Fischer et al., 2017 [48]	+	+									+	
Gebert et al., 2018 [29]	+	+	+	-	-	+	+	+	-	-	-	6
Häggland et al., 2005a[16]	+	+	-	+	-	+	+	+	+	-	+	8
Hägglund et al., 2006[17]	+	+	-	+	-	+	+	+	+	-	+	8
Hägglund et al., 2007a49	+	+	+	-	-	+	+	+	+	-	+	8
Hägglund et al., 2009 [50]	+	+	+	-	-	+	+	+	+	-	+	8
Hägglund et al., 2013[51]	+	+	+	-	-	+	+	+	-	+	+	8
Hägglund et al., 2016 [19]	+	+	+	-	-	+	+	+	-	-	+	7
Hammes et al., 2014 [52]	+	+	+	-	-	+	+	-	+	-	+	7
Hawking et al.,1999 [53]	+	+	-	-	+	+	+	+	+	-	+	8
Herrero et al., 2014 [54]	+	+	+	-	-	+	+	+	+	-	-	7
Jones et al., 2019 [55]	+	+	+	+	-	+	+	+	+	-	+	9
Kekelekis et al., 2023 [30]	+	+	+	+	-	+	+	+	+	-	+	9
Kordi et al., 2011 [56]:	+	+	+	-	-	+	+	-	-	-	+	6
Lee et al., 2014 [57]	+	+	+	-	-	+	+	+	+	-	+	8
Mallo et al., 2011 [58]	+	+	+	-	-	+	+	+	+	-	+	8
Martins et al., 2022 [59]	+	+	+	-	-	+	+	+	+	-	+	8
Morgan et al., 2001 [60]	+	+	+	-	-	+	+	+	-	-	+	7
Murphy et al., 2012 [61]	+	+	+	-	-	+	+	+	-	-	+	7
Nogueira et al., 2017 [28]	+	+	+	-	-	+	+	+	+	-	-	8
Noya Salces et al., 2014b [62]	+	+	+	+	-	+	+	+	+	-	+	9
Noya Salces et al., 2014a [63]	+	+	+	+	-	+	+	+	+	-	+	9
Parry and Drust et al., 2006[64]	+	+	-	-	-	+	+	+	+	-	+	7
Reis et al., 2015 [65]	+	+	-	-	-	+	+	+	+	-	+	8
Roe et al., 2018 [66]	+	+	+	-	-	+	+	+	+	-	+	8
Shalaj et al., 2016 [67]	+	+	+	-	+	+	+	+	+	-	+	9
Sousa et al., 2012 [26]	+	+	-	+	-	+	+	+	+	-	+	8
Stubbe et al., 2015 [68]	+	+	+	+	+	+	+	+	+	+	+	11
Waldén et al., 2005a [69]	+	+	-	-	+	+	+	+	+	-	+	8
Waldén et al., 2005b [4]	+	+	+	-	-	+	+	+	+	-	+	8
Waldén et al., 2007 [70[	+	+	-	+	-	+	+	+	+	-	+	8
Van Beijsterveldt et al., 2012 [27]	+	+	+	-	-	+	+	+	+	-	+	8

**Mean value ± SD**	+	**8.55±0.69**

The numbers of the columns corresponded to the following items of the STROBE scale: 1. Describes the setting or participating locations; 2. Describes relevant dates (period of recruitment, exposure, follow-up, data collection); 3. Provides statement concerning institutional review board approval and consent; 4. Gives the inclusion and exclusion criteria; 5. Describes injury history; 6. Describes methods of follow-up; 6. Data sources/measurement; 7. Provides a definition of injury; 8. Verifies injury by an independent medical professional; 9. Classifies injury (severity, location and type of injury); 10. Indicates the number of participants with missing data and explain how this was addressed; 11. Measures and presents exposure data.

Table S6 Risk of bias assessment of the studies (Newcastle-Ottawa scale; n=46).

Study: Author, Year	1	2	3	4	5	6	7	8	Scoring*/ Risk
Arnason et al., 2005 [37]	-	*	*	*	*	*	*	*	7/Low
Aus der Fünten et al., 2023 [23]	*	*	*	*	*	*	*	*	8/Low
Bayne et al., 2018 [39]	-	*	*	*	*	-	*	*	6/Moderate
Brito et al., 2012 [25]	*	*	*	-	*	-	*	*	6/Moderate
Dvorak et al., 2011 [40]	-	*	*	-	*	*	-	*	5/High
Dupont et al., 2010; France [41]	*	*	*	*	*	*	*	*	8/Low
Eirale et al., 2010 [42]	*	*	*	*	*	*	*	-	7/Low
Eirale et al., 2013[43]	*	*	*	*	*	*	*		7/Low
Ekstrand et al., 1990 [44]	*	*	*	-	*	*	-	*	6/Moderate
Ekstrand et al., 2004a [45]	*	*	*	-	*	-	*	-	5/High
Ekstrand et al., 2004b [46]	-	*	*	*	*	-	*	*	6/Moderate
Ekstrandet al., 2011a [47]	*	*	*		*	*	*	*	7/Low
Ekstrandet al., 2011b [14]	*	*	*	-	*	*	*	*	7/Low
Fischer et al., 2017 [48]	*	*	*	-	*	*	*	-	6/Moderate
Gebert et al., 2018 [29]	*	*	*	-	*	*	-	-	5/High
Häggland et al., 2005a[16]	*	*	*	*	*	*	*		7/Low
Hägglund et al., 2006[17]	*	*	*	*	*	*	*	*	8/Low
Hägglund et al., 2007a49	*	*	*	*	*	*	*	-	7/Low
Hägglund et al., 2009 [50]	*	*	*	*	*	*	*	-	7/Low
Hägglund et al., 2013[51]	*	*	*	-	*	-	*	-	5/High
Hägglund et al., 2016 [19]	*	*	*	-	*	-	*	-	5/High
Hammes et al., 2014 [52]	*	*	*	-	*	*	*	-	6/Moderate
Hawking et al.,1999 [53]	*	-	*	*	*	*	*	*	7/Low
Herrero et al., 2014 [54]	*	*	*	*	*	-	*	*	7/Low
Jones et al., 2019[55]	*	*	*	-	*	*	*	-	6/Moderate
Kekelekis et al., 2023 [30]	*	*	*	*	*	*	*	*	8/Low
Kordi et al., 2011[56]:	*	*	*	-	*	-	*	-	5/High
Lee et al., 2014 [57]	*	*	*	-	*	*	*	-	6/Moderate
Mallo et al., 2011 [58]	*	*	*	-	*	*	*	*	7/Low
Martins et al., 2022 [59]	*	*	*	-	*	-	*	*	6/Moderate

Morgan et al., 2001[60]	*	*	*	-	*	-	*	*	6/Moderate
Murphy et al., 2012 [61]	*	*	*	-	*	*	*	*	7/Low
Nogueira et al., 2017[28]	*	*	*	*	*	*	*	-	7/low
Noya Salces et al., 2014b [62]	*	*	*	*	*	*	*	*	8/Low
Noya Salces et al., 2014a [63]	*	*	*	*	*	*	*	*	8/Low
Parry and Drust et al., 2006[64]	*	*	*-	-	*	-	-	*	5/High
Reis et al., 2015 [65]	*	*	*	*	*	*	*	*	8/Low
Roe et al., 2018 [66]	*	*	*	-	*	-	*	*	6/Moderate
Shalaj et al., 2016[67]	*	*	*	-	*	*	*	*	7/Low
Sousa et al., 2012 [26]	*	*	*		*	*	*	*	7/Low
Stubbe et al., 2015 [68]	*	*	*	-	*	*	*	*	7/Low
Waldén et al., 2005a [69]	*	*	*	-	*	-	*	*	6/Moderate
Waldén et al., 2005b[4]	*	*	*	*	*	*	-	*	7/Low
Waldén et al., 2007 [70[	*	*	*	-	*	*	-	*	7/Low
4Van Beijsterveldt et al., 2012 [27]	*	*	*	*	*	*	*	*	8/Low
**Mean value ±SD**									**6.63±0.8**

1. Study setting (Description type of football players; location and period); 2. Definition of injury; 3. Representativeness of exposed cohort; 4. Exposure defining and measuring; 5. Demonstration that the outcome of interest was not present at the beginning of the study; 6. Evaluation of the results; 7. There was a long enough follow-up for the results to occur; 8. Adequacy of cohort monitoring; *Number of items with risk low of BIAS

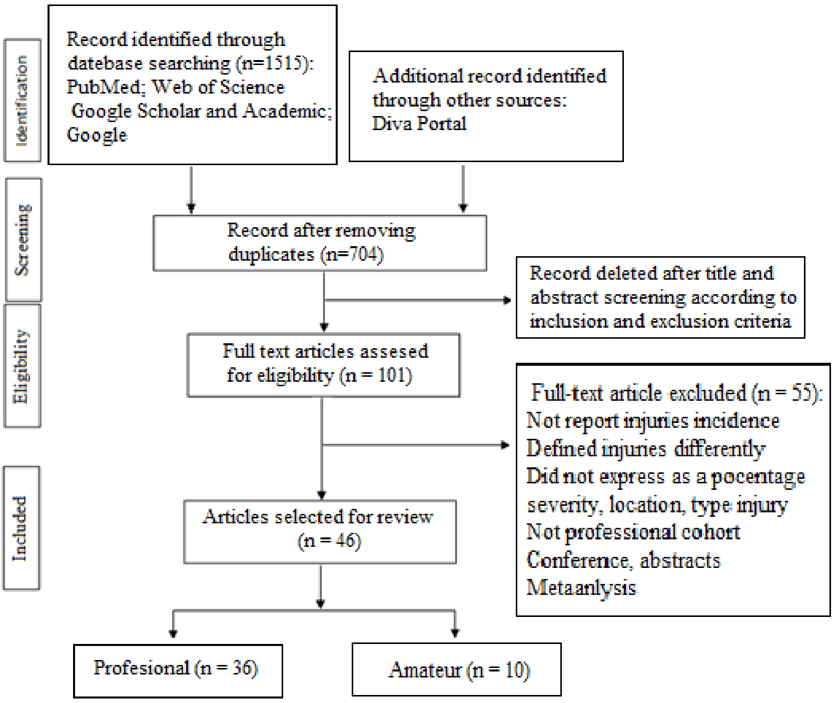

Figure S1. PRISMA flow chart
